# Parenting stress characteristics and related factors among female clinical nurses working a shift schedule raising preschool children: a latent profile analysis

**DOI:** 10.3389/fpubh.2026.1938684

**Published:** 2026-10-14

**Authors:** Qingfang Yao, Jianghong Yu, Hu Jiang, Jianlei Xie, Yunxiu Yang, Bo Yang

**Affiliations:** 1 Chongqing Mental Health Center, Chongqing, China; 2 The Third Affiliated Hospital of Zunyi Medical University (The First People's Hospital of Zunyi), Zunyi, Guizhou, China

**Keywords:** associated factors, clinical nurses, cross-sectional study, latent profile analysis, parenting stress

## Abstract

**Objectives:**

To investigate the underlying characteristics of parenting stress among clinical nurses in Guizhou Province, China, who work shifts and raise preschool children, and the factors influencing this stress.

**Design:**

Cross-sectional study.

**Setting:**

The public hospitals in Guiyang and Zunyi city, Guizhou Province, China.

**Methods:**

From 1 May to 31 December 2024, a total of 1,140 female clinical nurses were enrolled. A total of 1,108 valid questionnaires were retrieved, representing a response rate of 97.19%. Data collection was conducted using the parenting stress scale for clinical nurses and a demographic questionnaire. Identifying distinct characteristics of parenting stress among female clinical nurses through latent profile analysis, and determining factors associated with parenting stress among female clinical nurses using univariate analysis and multinomial logistic regression.

**Results:**

The Parenting stress experienced by female clinical nurses can be categorized into four distinct profiles: low-stress profile (10.11%), moderate-stress profile (26.81%), high-stress profile (52.26%) and shift-work-dominant profile (10.83%). The results of the multinomial logistic regression indicate that nurses’ parenting stress is significantly associated with the following factors: night shift (per month) (6 ~ 10, 11 ~ 15, 16~), family atmosphere (average or poor), child’s physical condition (unhealthy), number of children (2and 3~), parenting helper (occasionally and never), department atmosphere (average or poor).

**Conclusion:**

This study employed latent profile analysis to identify four distinct subgroups, revealing that night shifts (per month), family atmosphere, the child’s physical condition, number of children, parenting helper, and department atmosphere are factors influencing nurses’ parenting stress. These findings underscore the necessity of developing strategies to address nurses’ parenting stress in order to promote their well-being.

## Introduction

Against the backdrop of profound transformation within global healthcare systems, the stability and sustainable development of nursing human resources have become pivotal factors influencing healthcare quality. As the backbone of healthcare delivery, the physical and mental wellbeing of clinical nurses not only impacts their individual professional advancement but also directly affects patient safety and the quality of care delivered (1). In recent years, with the full implementation of China’s three-child policy and the accelerated pace of population aging, clinical nursing staff face the dual pressures of professional responsibilities and family obligations. Statistical data indicates that the total number of registered nurses in China has surpassed five million, with women constituting 98.2% of this workforce. Nurses of childbearing age form the core of the nursing workforce, accounting for over 60% of the total (2). This group must juggle demanding clinical work with increasingly complex childcare responsibilities, creating a unique pattern of work–family conflict.

Parenting Stress was first proposed by psychologist Abidin in 1976, referring to the pressure parents encounter while fulfilling their parental responsibilities, primarily stemming from both the child and the parent (3). It is influenced by multiple factors, including the characteristics of the child and parents, the family and social environment, parent–child relationships, and interpersonal relationships (4). These factors may Impact shifts in psychological experiences during child-rearing, constituting a distinct form of stress.

Female nurses of childbearing age, as the backbone of clinical nursing, exhibit distinct occupational imprints on their parenting stress. They not only face intense workloads, irregular schedules, and persistent emotional strain, but must also navigate multiple developmental demands including career advancement, research commitments, and clinical teaching responsibilities. The implementation of China’s three-child policy aimed to alleviate parenting stress through supporting measures, thereby boosting fertility rates. However, the prevailing economic pressures, childcare challenges, and career development constraints faced by most families remain key factors limiting the policy’s effectiveness (5). Moreover, the evolution of modern parenting concepts has expanded parental responsibilities beyond basic care to encompass multidimensional demands such as educational nurturing and emotional companionship, fostering societal expectations of “intensive parenting.” The period from 3 to 6 years marks a critical stage in a child’s character formation, with heightened emotional demands placing increasing strain on caregivers. Prolonged accumulation of such pressures may lead to emotional exhaustion among nurses, diminished parenting efficacy, and reduced work engagement. This, in turn, compromises clinical judgment and procedural safety, thereby constituting the phenomenon of dual occupational-parental burden (6).

The parenting stress experienced by female clinical nurses has been the subject of attention from numerous scholars both domestically and internationally, though the depth and breadth of research vary considerably. Overseas studies commenced earlier and have established a relatively systematic theoretical framework (4). Multiple studies indicate that parenting stress among nursing staff generally falls at a moderately high level. For instance, a cross-sectional study of nurses in the South Korean capital who were raising children under the age of five revealed that shift work and high-intensity workloads were the primary challenges contributing to parenting stress in this group, and that parenting stress was significantly negatively correlated with their job stability (7). In a study focusing primarily on female nurses in their thirties, the level of parenting stress was found to be moderate or higher (8). Parenting stress is significantly positively correlated with nurses’ work-related fatigue, intention to leave, and risk of medical errors (9). Domestic research in this field, though relatively recent in inception, has developed rapidly in recent years. With the implementation of the three-child policy and growing public emphasis on family nurturing, the issue of parenting stress among clinical nurses has become increasingly prominent. Research indicates that Chinese nurses’ parenting stress levels are closely linked to occupational role conflict. For instance, a survey of 60,211 nurses of childbearing age in China revealed an average occupational development score of (123.02 ± 19.67) points and a work–family conflict score of (39.20 ± 14.52) points, both at moderate levels, suggesting a serious problem (10). Another study found that nurses in specialized departments such as emergency departments experience significantly higher levels of parenting stress than their counterparts in other departments, owing to the distinctive pace and intensity of their work (11). However, research also suggests that there exists a significant bidirectional enhancement between nurses’ professional roles and their family lives (12).

Regarding measurement tools, the Parenting Stress Scale for Clinical Nurses (PSSCN), developed in 2021 by Professor Lee of South Korea and colleagues, is currently widely employed. This scale has been applied in research examining the relationship between clinical nurses’ parenting stress and workload, occupational stress, and social support, demonstrating good reliability and validity (13). Chinese scholar Zeng adapted it into Chinese in 2023, and it has since gained widespread application in China (14).

Although parenting stress among nurses has been increasingly studied, most research treats it as a continuous variable, neglecting its qualitative heterogeneity and potential subgroups. Exploration across different stress types remains scarce, limiting both mechanistic understanding and intervention development. Thus, alternative approaches that capture latent stress patterns are needed. Latent Profile Analysis (LPA) is a person-centered method that identifies subgroups based on observed variable response patterns (15). Using statistical fit indices, it provides a robust alternative to subjective clustering (16), ensuring clear subgroup differentiation. While this approach offers valuable insights into parenting stress heterogeneity, its application to clinical nurses’ parenting stress remains unexplored.

Given the value of understanding parenting stress heterogeneity among clinical nurses, this study addressed two research questions: (1) What distinct parenting stress profiles exist among clinical nurses working shift schedules and raising preschool children in Guizhou, China? and (2) What factors are associated with profile membership? To answer these questions, we used latent profile analysis to identify subgroups and examine related factors.

## Methods

### Design

This cross-sectional study employed an online survey to collect data, utilizing convenience sampling. This method selects readily accessible participants under resource-constrained conditions, aiming to enhance the feasibility and efficiency of the research. Both the study design and results reporting adhered to the relevant specifications outlined in the Strengthening the Reporting of Observational Studies in Epidemiology (17).

### Sample size estimation

Scholars (18) recommend a minimum sample size of 300 cases for LPA analysis to avoid bias when identifying smaller latent profiles. Furthermore, to ensure the robustness of subsequent multivariate analysis results, this study adhered to the general principle requiring the number of sample observations to be at least five times the number of variables (19). Considering a 20% invalid questionnaire return rate, calculations indicated a minimum requirement of N = (14 + 19) × 5 / (1–20%) ≈ 206 participants. The final valid sample size of 1,108 cases met the aforementioned requirements.

### Participants

From May to December 2024, nurses in Guiyang and Zunyi cities, Guizhou Province, were invited to participate in this survey. Inclusion criteria: ① Female nurses working on clinical frontline positions undertaking periodic night shifts; ② Raising at least one preschool-aged child (3–6 years old); ③ Voluntary participation in the survey. Exclusion criteria: ① Nurses in clinical support roles; ② Nurses in probationary, internship, or training positions; ③ Nurses absent from duty during the survey period due to leave or other reasons; ④ Single mothers.

## Measurements

### Demographic questionnaire

Researchers compiled a demographic information questionnaire encompassing fundamental demographic data such as age, education, professional title, employment types, nursing experience (years), night shift (per month), nursing patients (per day), average monthly income, family atmosphere, child’s physical condition, number of children, parenting helper, department, department atmosphere.

### Parenting stress scale for clinical nurses (PSSCN)

The PSSCN was developed in 2021 by Professor Lee in South Korea (13). This study employed the Chinese version of the scale translated by Zeng (14), which has undergone reliability and validity testing alongside cultural adaptation in China, demonstrating sound reliability and validity. The scale comprises four dimensions: psychological burden, physical and mental fatigue, shift work, and work environment, total 19 items. A four-point rating scale was employed, with responses ranging from ‘Strongly Disagree’ to ‘Strongly Agree’ scored as 1 to 4 points, respectively. The total score ranges from 19 to 76 points, with higher scores indicating greater nursing burden. The content validity index ranged from 0.88 to 0.96, with criterion-related validity at 0.622. In this study, the Cronbach’s α coefficient for this scale was 0.826, and a test–retest reliability of 0.888. Confirmatory factor analysis validated the scale’s structural validity, with all model indices showing good fit. This study utilized the scores on each dimension of the scale as observed variables for LPA, in order to identify latent subgroups of parenting stress.

### Data collection

This study employed an anonymous data collection method. Prior to the formal survey, a pilot study was conducted in February 2024 involving 30 female clinical nurses of childbearing age to assess the questionnaire’s comprehensibility and technical feasibility, with a minimum response time of 100 s (pilot data were excluded from formal analysis). The questionnaire was distributed online via the ‘Wenjuanxing’,^1^ with content jointly reviewed by two researchers. Concurrently, an electronic poster containing the questionnaire link and QR code was produced, clearly outlining inclusion and exclusion criteria for participants. The survey was conducted in Guiyang and Zunyi cities. Researchers liaised with nursing managers at local healthcare institutions to assist in distributing posters. Clinically active female nurses of childbearing age voluntarily participated and submitted questionnaires via the poster links or QR codes. Following data collection, two researchers manually verified the returned data, excluding invalid responses (e.g., questionnaires completed in ≤100 s).

### Statistical analyses

This study employed Mplus 8.4 software to conduct latent profile analysis (LPA) of parenting stress among female clinical nurses of childbearing age. By progressively increasing the number of latent profiles (from one to five categories), five models were established for comparison. Model fit was assessed using the following indices: Akaike Information Criterion (AIC), Bayesian Information Criterion (BIC), and adjusted BIC (aBIC), where lower values indicate better model fit; entropy values closer to 1 denote higher classification accuracy (18). Concurrently, the Lo–Mendell–Rubin likelihood ratio test (LMR) and Bootstrap-based likelihood ratio test (BLRT) were employed to compare adjacent models. A *p*-value < 0.05 indicated the current model was significantly superior to the preceding one (20). When determining the optimal number of profiles, in addition to the aforementioned statistical metrics, we comprehensively considered model parsimony, the proportion of samples allocated to each profile (no less than 5% of the total sample), and their theoretical significance and interpretability within the practical research context. This ensured that the extracted latent profiles possessed substantive differentiation and practical value.

Data statistical analysis was conducted using SPSS 27.0 software. Count data were presented as frequencies and percentages or rates. Intergroup comparisons employed chi-square tests or rank-sum tests. Multinomial logistic regression was performed, using LPA classification outcomes as the dependent variable and variables demonstrating statistical significance in univariate analysis as independent variables. The significance level was set at α = 0.05.

### Ethics considerations

The Ethics Committee of the Third Affiliated Hospital of Zunyi Medical University approved this study (2023-1-255).

## Results

### Common method bias test

This study employed self-report questionnaires for data collection. To mitigate potential common method bias, participants were explicitly informed of the questionnaires’ anonymity and confidentiality, with data usage restricted solely to academic research, thereby procedurally reducing sources of bias. Furthermore, Harman’s single-factor test was employed to assess method bias. The analysis extracted three unrotated factors. The first factor explained 21.359% of the variance, falling below the 40% threshold for significant concern. This indicates that the data in this study did not exhibit severe common method bias.

### Demographic characteristics

This study invited 1,140 nurses to participate in the survey, of whom 13 declined to take part, 9 submitted responses containing logical errors, and 10 completed the questionnaire in an insufficient timeframe (≤100 s). Ultimately, 1,108 valid questionnaires were retrieved, yielding a valid response rate of 97.19%. Valid respondents were categorized into three age groups: 20–29 years (*n* = 246), 30–39 years (*n* = 711), and ≥40 years (*n* = 151).

### Model fit indices of LPA

This study utilized the scores across the various dimensions of the PSSCN as observed variables and employed LPA to identify latent categories of parenting stress among clinical nurses. Model fitting was performed by gradually increasing the number of profiles; the fit indices for each model are shown in Table 1. The results show that, as the number of cross-sections increases, the AIC, BIC and aBIC values gradually decrease, reaching their lowest values for the four-profile model; however, for the five-profile model, these values rise rather than fall, indicating that the four-profile model provides the best fit, its entropy value was 0.951, yielding the highest classification accuracy; the likelihood ratio test reached statistical significance; and the sample sizes for all four categories exceeded 5 per cent of the total sample, with a balanced distribution and clear theoretical interpretability for each profile. Based on the fit statistics, classification quality and theoretical soundness, the four-profile model was ultimately selected as the optimal solution.

**Table 1:** Fit statistics for profile structure (*n* = 1,108).

Model	AIC	BIC	aBIC	Entropy	LMRT (*p*)	BLRT (*p*)	Category probability
1	23308.066	23348.149	23322.739	—	—	—	1
2	21867.426	21932.560	21891.269	0.810	<0.001	<0.001	0.640/0.360
3	21032.648	21122.234	21065.061	0.891	0.0189	<0.001	0.143/0.408/0.449
4	20085.370	20225.659	20136.724	0.951	<0.001	<0.001	0.108/0.101/0.268/0.523
5	20320.639	20435.876	20362.823	0.904	0.0017	<0.001	0.101/0.223/0.153/0.422/0.101

AIC, Akaike Information Criterion; BIC, Bayesian Information Criterion; aBIC, adjusted BIC; LMRT, Lo Mendell-Ruben adjusted likelihood ratio test; BLRT, bootstrap likelihood ratio test.

Based on LPA results, this study identified four distinct characteristic profiles of parenting stress among clinical nurses. Profile 1 (C1), labeled the ‘Low-stress profile’, comprised nurses scoring low across all dimensions of parenting stress, with a total score of 20 (19, 26). This group included 112 nurses, representing 10.11% of the total sample. Profile 2 (C2) was categorized as the ‘moderate-stress profile’, with a total score of 39 (37, 43). It comprised 297 nurses, representing 26.81% of the sample. Factor Profile 3 (C3) was identified as the ‘high-stress profile’, with a total score of 55 (50, 57). This group comprised 579 nurses, representing 52.26% of the sample. Profile 4 (C4) was termed the ‘shift-work-dominant profile’, characterized by high scores on the shift work dimension and low scores on other dimensions. It comprised 120 nurses, representing 10.83% of the total sample. The LPA results are illustrated in Figure 1.

**Figure 1:**
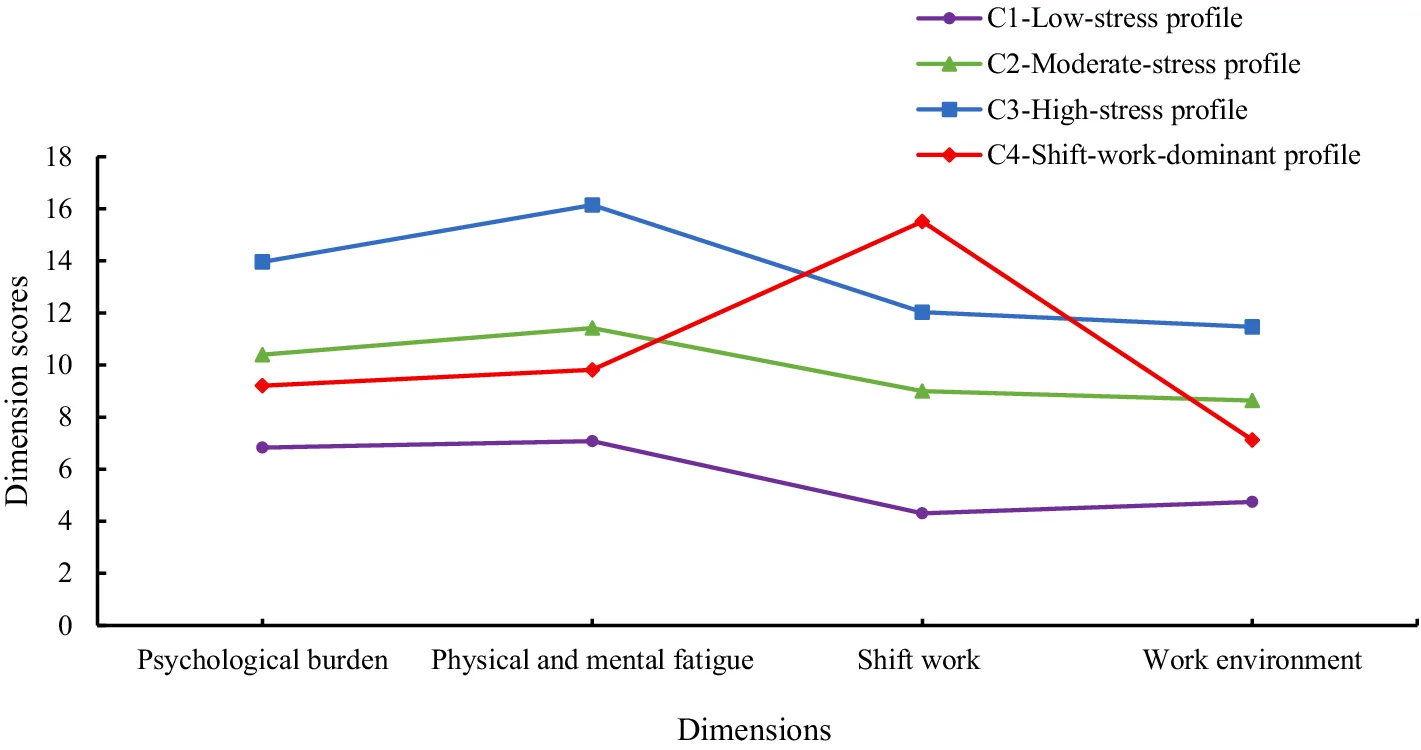
Estimated parenting stress dimension scores across the four latent profiles.

### Demographic and related characteristics of each profile

Following the identification of distinct clinical nursing parenting stress groups via the LPA, chi-square tests were employed to assess statistically significant differences in demographic characteristics across these groups. Results indicated statistically significant differences between groups for the following variables: night shifts (per month), nursing patients (per day), family atmosphere, child’s physical condition, number of children, parenting helper, and department atmosphere. No significant associations were observed for the remaining variables. Detailed results are presented in Table 2.

**Table 2:** Demographic and related characteristics of each profile (*n* = 1,108).

Variables	Low-stress profile (*n* = 112)	Moderate-stress profile (*n* = 297)	High-stress profile (*n* = 579)	Shift-work-dominant profile (*n* = 120)	χ2	*p* value
Age					7.460	0.280
20 ~ 29	28 (25.00)	59 (19.87)	138 (23.83)	21 (17.50)		
30 ~ 39	72 (64.29)	189 (63.64)	364 (62.87)	86 (71.67)		
40~	12 (10.71)	49 (16.50)	77 (13.30)	13 (10.83)		
Education					0.598	0.897
Associate degree	8 (7.14)	21 (7.07)	45 (7.77)	7 (5.83)		
Undergraduate degree and above	104 (92.86)	276 (92.93)	534 (92.23)	113 (94.17)		
Professional title					5.404	0.493
Junior-level	50 (44.64)	145 (48.82)	267 (46.11)	55 (45.83)		
Intermediate-level	57 (50.89)	132 (44.44)	285 (49.22)	62 (51.67)		
Senior-level	5 (4.46)	20 (6.73)	27 (4.66)	3 (2.50)		
Employment types					4.910	0.178
Non-permanent staff	82 (73.21)	199 (67.00)	425 (73.40)	81 (67.50)		
Permanent employee	30 (26.79)	98 (33.00)	154 (26.60)	39 (32.50)		
Nursing experience (years)					20.364	0.061
<5	30 (26.79)	52 (17.51)	151 (26.08)	29 (24.17)		
6 ~ 10	33 (29.46)	125 (42.09)	202 (34.89)	47 (39.17)		
11 ~ 15	26 (23.21)	52 (17.51)	106 (18.31)	21 (17.50)		
16 ~ 20	17 (15.18)	42 (14.14)	94 (16.23)	18 (15.00)		
21~	6 (5.36)	26 (8.75)	26 (4.49)	5 (4.17)		
Night shift (per month)					40.350	<0.001
1 ~ 5	57 (50.89)	134 (45.12)	198 (34.20)	22 (18.33)		
6 ~ 10	10 (8.93)	24 (8.08)	60 (10.36)	15 (12.50)		
11 ~ 15	17 (15.18)	57 (19.19)	109 (18.83)	33 (27.50)		
16~	28 (25.00)	82 (27.61)	212 (36.61)	50 (41.67)		
Nursing patients (per day)					23.856	0.005
<4	31 (27.68)	73 (24.58)	103 (17.79)	17 (14.17)		
4 ~ 8	22 (19.64)	51 (17.17)	111 (19.17)	23 (19.17)		
8 ~ 12	23 (20.54)	58 (19.53)	105 (18.13)	13 (10.83)		
12~	36 (32.14)	115 (38.72)	260 (44.91)	67 (55.83)		
Average monthly income					8.989	0.178
<5,000	73 (65.18)	165 (55.56)	328 (56.65)	56 (46.67)		
5–10,000	28 (25.00)	94 (31.65)	188 (32.47)	46 (38.33)		
10,000~	11 (9.82)	38 (12.79)	63 (10.88)	18 (15.00)		
Family atmosphere					39.847	<0.001
Great	100 (89.29)	226 (76.09)	371 (64.08)	72 (60.00)		
Average or poor	12 (10.71)	71 (23.91)	208 (35.92)	48 (40.00)		
Child’s physical condition					13.969	0.003
Healthy	108 (96.43)	271 (91.25)	504 (87.05)	101 (84.17)		
Unhealthy	4 (3.57)	26 (8.75)	75 (12.95)	19 (15.83)		
Number of children					42.201	<0.001
1	86 (76.79)	182 (61.28)	295 (50.95)	56 (46.67)		
2	20 (17.86)	101 (34.01)	252 (43.52)	49 (40.83)		
3~	6 (5.36)	14 (4.71)	32 (5.53)	15 (12.50)		
Parenting helper					36.652	<0.001
Always	66 (58.93)	133 (44.78)	190 (32.82)	37 (30.83)		
Occasionally	24 (21.43)	96 (32.32)	234 (40.41)	47 (39.17)		
Never	22 (19.64)	68 (22.90)	155 (26.77)	36 (30.00)		
Department					19.460	0.364
Internal medicine system	32 (28.57)	72 (24.24)	145 (25.04)	31 (25.83)		
Surgical system	26 (23.21)	77 (25.93)	163 (28.15)	28 (23.33)		
Gynecology and obstetrics system	13 (11.61)	41 (13.80)	46 (7.94)	17 (14.17)		
Pediatric system	21 (18.75)	46 (15.49)	100 (17.27)	19 (15.83)		
Outpatient and emergency system	15 (13.39)	37 (12.46)	73 (12.61)	13 (10.83)		
Critical care system	3 (2.68)	12 (4.04)	37 (6.39)	10 (8.33)		
Function department	2 (1.79)	12 (4.04)	15 (2.59)	2 (1.67)		
Department atmosphere					27.720	<0.001
Great	100 (89.29)	250 (84.18)	425 (73.40)	82 (68.33)		
Average or poor	12 (10.71)	47 (15.82)	154 (26.60)	38 (31.67)		

### Results of multinomial logistic regression

This study employed multinomial logistic regression, utilizing four latent profiles as dependent variables and incorporating significant univariate predictors including night shift, nursing patients, family atmosphere, and child’s physical condition. With Group C1 as the reference group, results indicate that clinical nurses experiencing an average or poor family atmosphere, having three or more children, and lacking parental support (never) were more likely to be classified into Group C2. Those working night shifts (16~), experiencing an average or poor family atmosphere, having three or more children, and receiving occasional or no parental support were more likely to belong to Group C3. Clinical nurses with night shifts (6 ~ 10, 11 ~ 15, 16~), family atmosphere (average or poor), child’s physical condition (unhealthy), number of children (2, 3~), parenting helper (occasionally, never), and department atmosphere (average or poor) were more likely to be assigned to Group C4. Detailed analysis results are presented in Table 3.

**Table 3:** Results of multinomial logistic regression.

Variables	C2 vs. C1			C3 vs. C1			C4 vs. C1		
	*β*	OR (95% CI)	*p*	*β*	OR (95% CI)	*p*	*β*	OR (95% CI)	*p*
Night shift (per month) (Ref: 1 ~ 5)									
6 ~ 10	−0.018	0.982 (0.433, 2.228)	0.966	0.482	1.620 (0.750,3.496)	0.219	1.265	3.544 (1.324,9.486)	0.012
11 ~ 15	0.321	1.379 (0.717, 2.652)	0.336	0.517	1.678 (0.889,3.167)	0.111	1.570	4.805 (2.12,10.889)	<0.001
16~	0.185	1.204 (0.649, 2.088)	0.510	0.672	1.959 (1.157,3.319)	0.012	1.404	4.073 (1.995,8.313)	<0.001
Nursing patients (per day) (Ref: <4)									
4 ~ 8	−0.022	0.978 (0.497, 1.926)	0.949	0.300	1.350 (0.701,2.597)	0.369	0.301	1.351 (0.555,3.287)	0.508
8 ~ 12	0.036	1.036 (0.533, 2.015)	0.916	0.234	1.264 (0.660,2.422)	0.480	−0.226	0.797 (0.307,2.072)	0.642
12~	0.216	1.242 (0.684, 2.254)	0.477	0.570	1.769 (0.990,3.160)	0.054	0.755	2.128 (0.982,4.611)	0.056
Family atmosphere (Ref: good)									
Average or poor	0.777	2.174 (1.100, 4.296)	0.025	1.126	3.083 (1.604,5.927)	0.001	1.178	3.248 (1.538,6.858)	0.002
Child’s physical condition (Ref: healthy)									
Unhealthy	0.705	2.023 (0.676, 6.055)	0.208	0.954	2.595 (0.899,7.495)	0.078	1.161	3.195 (1.004,10.164)	0.049
Number of children (Ref: 1)									
2	0.021	1.021 (0.373, 2.793)	0.968	0.292	1.339 (0.525,3.413)	0.541	1.179	3.252 (1.138,9.291)	0.028
3~	0.872	2.391 (1.376, 4.154)	0.002	1.319	3.741 (2.195,6.376)	<0.001	1.366	3.921 (2.056,7.481)	<0.001
Parenting helper (Ref: Always)									
Occasionally	0.389	1.475 (0.830,2.622)	0.185	0.821	2.273 (1.311,3.941)	0.003	1.004	2.730 (1.358,5.49)	0.005
Never	0.577	1.781 (1.031,3.076)	0.038	1.000	2.717 (1.607,4.597)	<0.001	0.986	2.682 (1.379,5.215)	0.004
Department atmosphere (Ref: good)									
Average or poor	0.198	1.218 (0.604, 2.457)	0.581	0.645	1.907 (0.982,3.702)	0.057	0.859	2.361 (1.101,5.064)	0.027

Ref, reference.

## Discussion

This study employed latent profile analysis (LPA) to identify heterogeneous characteristics of parenting stress among clinical nurses. Surveying 1,108 clinical nurses revealed four latent profiles: the low-stress profile, moderate-stress profile, high-stress profile, and shift-work-dominant profile. To our knowledge, this represents the first investigation using LPA to explore heterogeneous groups of parenting stress among clinical nurses. Compared to traditional variable-centered analytical approaches, LPA identifies subgroups based on individuals’ response patterns across multiple dimensions, effectively capturing intrinsic heterogeneity within groups. This heterogeneity may be closely associated with factors such as nurses’ personal characteristics, work environments, and family circumstances (21). The findings provide novel empirical evidence regarding the distribution characteristics and group structure of nursing parenting stress, establishing a population segmentation foundation for subsequent precise identification and categorized interventions.

### Subgroup characteristics of parenting stress among clinical nurses

In this survey, the C3 accounted for 52.26%, indicating that over half of clinical nurses experience elevated levels of parenting stress. This suggests that, due to the specific nature of the nursing profession, nurses’ family roles may be compromised, a finding consistent with the results of most studies (21, 22). The C3group exhibited elevated scores across all parenting stress dimensions, demonstrating comprehensive stress characteristics. This indicates that nurses in this subgroup face systemic challenges in parenting task burdens, quality of parent–child relationships, perceived spousal support, and role balancing capabilities. The C2 constituted 26.81%, exhibiting intermediate stress levels. These nurses may possess some coping resources but have yet to develop stable psychological protective mechanisms. The C1 accounts for 10.11 per cent, which suggests that, given the current professional environment and family structures, there are relatively few nurses who are not burdened by the pressures. Notably, while the C4 scored low across other parenting stress dimensions, it exhibited significantly elevated scores on the shift work dimension, demonstrating elevated stress within a single dimension. Comprising 10.83% of the total sample, this group’s stress originates predominantly from work scheduling itself, rather than inadequate parenting skills or insufficient family support. This finding extends the boundaries of traditional parenting stress research, which has predominantly treated parenting stress as an endogenous psychological variable within the family domain (23, 24). This study confirms that occupational factors, particularly shift work arrangements, constitute a key stressor independent of familial variables. Consequently, nursing parenting stress is not merely a ‘parenting dilemma’ but rather a product of ‘institutional mismatch between work and family systems’.

### The cumulative effects of institutional factors in the workplace

Occupational systemic factors constitute one of the core variables distinguishing different parenting stress profiles. Using the C1 as a reference, within C4, each frequency of night shifts exhibits a significant predictive effect, peaking at 11–15 shifts per month. Within C3 night shifts (≥16) emerge as a significant risk factor. This indicates that frequent night shifts may be linked to the pressures of parenting. Night shifts disrupt nurses’ circadian rhythms, causing their working hours to clash with their family schedules, thereby hindering the practice of ‘attuned parenting’ (25, 26). Compounded by society’s more stringent childcare expectations placed upon mothers compared to fathers, this imposes gendered pressures. Concurrently, the high intensity of nursing work exacerbates physical and mental exhaustion, with occupational burnout spilling over into the home. This readily damages parent–child relationships, further deepening parenting difficulties (27). It is worth noting that the frequency of night shifts was not included in the regression model for C2, suggesting that the spillover of workplace stress into family life may be subject to a “threshold” effect (28). Below this threshold, nurses can maintain a balance of stress through individual adaptation and family buffer mechanisms; once this threshold is exceeded, the intrusion of workplace stress into family life transforms into systemic stress. Furthermore, another occupational factor, nursing patients (per day), though not included in the regression equation. This suggests that whether it is a predictor of childcare-related stress among nursing staff requires verification through research involving a larger sample size.

Given that this study found night shift frequency exceeding six shifts per month significantly increases the risk of belonging to the C4, it is recommended that the upper limit for monthly night shifts for female nurses during the child-rearing period be set at six. Concurrently, a predictable and negotiable flexible scheduling system should be implemented. Nurses should be permitted to receive their shift schedules 2 weeks in advance, with a negotiation channel provided for those with special needs, such as young children or children with poor health. Concurrently, individual workload should be reduced through measures such as increasing staffing levels and optimizing processes.

### The multifaceted impact of family structural factors

Family factors demonstrated consistent predictive effects in this study, with effect sizes generally exceeding those of occupational factors within the model. This suggests the family constitutes the primary setting for the generation of parenting stress and represents a key target for intervention.

Number of children emerged as the significant variable distinguishing all C3s from the C1 group. Compared to nurses with one child, those with three or more children exhibited a 2.391-fold increased risk of belonging to the C2, a 3.741-fold increased risk of belonging to the C3, and a 3.921-fold increased risk of belonging to the C4. The number of children directly reflects the aggregate demand for family nurturing. When this demand exceeds the family’s resource capacity, parenting stress emerges (29). Notably, having two children also presented a significant risk in the C4, though not reaching statistical significance in the C3. This suggests that under the cumulative strain of shift work, the resource-squeeze effect in two-child families may manifest earlier.

In addition to the number of children, the children’s health status also emerged as an important predictive factor. Compared with nurses whose children were healthy, nurses whose children were in poor health had a 3.195-fold increased risk of being classified as C4. This finding is consistent with the core conclusions of studies on families caring for children with chronic illnesses: children’s health problems not only place an additional burden on caregivers but also trigger persistent anxiety in mothers (29–31). Therefore, for nurses working rotating night shifts, the conflict between the demands of their profession and their children’s health needs constitutes a unique source of stress.

The absence of a parenting helper constitutes another core risk factor. This concept extends beyond mere division of household labor, representing a social determinant of nurses’ mental health. When familial support is inadequate, spousal involvement limited, or market services fail to meet childcare needs, nurses must navigate trade-offs between professional and maternal roles. Deficiencies in fulfilling either role intensify pressure on the other (32).

### The synergistic shaping of family and departmental atmospheres

The family atmosphere demonstrated a consistently significant predictive effect throughout this study. This finding indicates that parenting stress stems not only from objective circumstances but also from individuals’ subjective perceptions of their environment. It extends organizational climate research from the workplace to the family setting, validating Zhou’s research conclusions (31). When family atmosphere is harmonious, emotional support and understanding from spouses significantly buffer the emotional exhaustion arising from childcare. Conversely, when family atmosphere is strained, the home environment—which should be nurturing—transforms into a source of pressure. Nurses then become trapped in a bidirectional negative cycle of ‘work burnout and family conflict’ (4), further exacerbating their parenting stress.

In contrast to family atmosphere, department atmosphere (average or poor) exhibited significant predictive effects only within the C4. Shift work systems can diminish nurses’ participation in family life, significantly increasing their reliance on workplace support systems. When departmental atmosphere is poor, this amplifies role conflict risks (33). Conversely, a harmonious departmental atmosphere encourages nurses to confidently request flexible arrangements or seek emergency cover from colleagues when faced with sudden family demands. This reduces nurses’ emotional burden, enabling them to conserve more psychological resources for family commitments (34).

Consequently, interventions at the atmosphere level should concurrently target both the ward and the family sphere. At the ward level, ward atmosphere development should be integrated into nursing managers’ performance appraisal systems. Leadership training should be conducted, focusing on communication skills, conflict mediation, and emotional support. Regular ward atmosphere diagnostic surveys should be implemented, with targeted interventions for wards consistently scoring low. The hospital should concurrently organize events such as Family Open Days and Family Appreciation Gatherings to enhance family members’ understanding and recognition of the nursing profession. Develop psychological education materials targeted at nurses’ spouses to improve their cognitive understanding of the nursing profession and strengthen their willingness to provide support.

This study found no significant differences in factors such as age, associate degree, professional title, employment types, nursing experience, average monthly income, or department across different parenting stress profiles, potentially due to the high degree of homogeneity within the sample. For instance, nurses aged 30–39, holding undergraduate degrees or higher, at junior or intermediate levels, and non-permanent staff constituted a substantial proportion. The limited range of variable variation inherently reflects the structural realities of China’s nursing workforce: high educational attainment, youthfulness, and non-permanent status. Parenting stress has thus become a pervasive occupational challenge transcending traditional stratification dimensions. Future research should explore these variables through large-scale and longitudinal studies.

### Limitations

This study has the following limitations. Firstly, the cross-sectional design is unable to capture dynamic changes and makes it difficult to establish causal relationships between variables. Secondly, this study employed convenience sampling, and the sample was drawn exclusively from hospitals within Guizhou Province; although it covered hospitals of different levels and departments, its representativeness remains limited, and caution is required when generalizing the findings. Thirdly, all variables were self-reported, which may introduce bias. Fourthly, this study included only shift-working nurses; while this design helps to reveal the specific impact of shift work on parenting stress, it also limits the generalisability of the findings to non-shift-working groups. Fifthly, this study excluded single mothers; although this was done for methodological reasons to control for confounding effects of family structure, it restricts the generalisability of the findings to single-parent families.

## Conclusion

This study employed latent profile analysis to identify parenting stress subgroups among clinical nurses. Four distinct profiles were identified: low, moderate, and high-stress profiles, along with a shift work-dominated profile., and identified ‘night shifts’, ‘family atmosphere’, ‘the child’s physical condition’, ‘number of children’, ‘parenting support’ and ‘departmental atmosphere’ as associated factors. These findings reveal the heterogeneous nature of parenting stress within this group and suggest that hospitals should develop tailored intervention programs targeting different subtypes and their associated factors, thereby effectively alleviating the parenting stress experienced by nurses.

## Data Availability

The raw data supporting the conclusions of this article will be made available by the authors, without undue reservation.

## Footnotes

1
www.wjx.cn

## References

[1] Kohnen D De Witte H Schaufeli WB Dello S Bruyneel L Sermeus W. What makes nurses flourish at work? How the perceived clinical work environment relates to nurse motivation and well-being: a cross-sectional study. Int J Nurs Stud. (2023) 148:104567. doi: 10.1016/j.ijnurstu.2023.104567, 37837704

[2] National Health Commission. National nursing development plan (2021–2025). Chin Nurs Manag. (2022) 22:801–4. doi: 10.3969/j.issn.1672-1756.2022.06.001

[3] Chavez AE. Leaders in the history of clinical child and adolescent psychology past presidents series: Richard Abidin (1989). J Clin Child Adolesc Psychol. (1989) 54:405–8. doi: 10.1080/15374416.2025.2516465 40631975

[4] Fang Y Luo J Boele M Windhorst D van Grieken A Raat H. Parent, child, and situational factors associated with parenting stress: a systematic review. Eur Child Adolesc Psychiatry. (2024) 33:1687–705. doi: 10.1007/s00787-022-02027-1, 35876894 PMC11211171

[5] Tatum M. China's three-child policy. Lancet. (2021) 397:2238. doi: 10.1016/s0140-6736(21)01295-2, 34119051

[6] Loth KA Ji Z Wolfson J Fisher J Berge J Neumark-Sztainer D. Momentary predictors of a broad range of food parenting practices within a population-based sample of parents of preschool-aged children. Front Public Health. (2022) 10:944734. doi: 10.3389/fpubh.2022.944734, 36726615 PMC9885496

[7] Hwang M Lee N Lee G. How parenting-related characteristics influence parenting stress among nurses with young children in the Seoul metropolitan area, South Korea: a cross-sectional study. BMC Nurs. (2025) 24:1052. doi: 10.1186/s12912-025-03643-3, 40790585 PMC12337397

[8] Jung YE Sung MH. Do parenting stress, work-family conflict, and resilience affect retention intention in Korean nurses returning to work after parental leave?: a cross-sectional study. Korean J Women Health Nurs. (2022) 28:18–26. doi: 10.4069/kjwhn.2022.01.07 PMC9334207 36312046

[9] Li LZ Yang P Singer SJ Pfeffer J Mathur MB Shanafelt T. Nurse burnout and patient safety, satisfaction, and quality of care: a systematic review and meta-analysis. JAMA Netw Open. (2024) 7:e2443059. doi: 10.1001/jamanetworkopen.2024.43059 PMC11539016 39499515

[10] Ma XL Shen YZ Huang MG Huang YL. A study on the current status and influencing factors of career development among nurses of childbearing age in China. J Nurs Manag. (2022) 22:159–62, 67. doi: 10.3969/j.issn.1671-315x.2022.03.002

[11] Chen Y. Logistic analysis of the current status and influencing factors of work-family conflict among emergency department nurses. Chin J Health Stat. (2024) 41:893–4. doi: 10.11783/j.issn.1002-3674.2024.06.018

[12] Li XY Yu GZ Zhu S Han LZ Xiong XJ Lin T. A qualitative study on work-family gain experiences among paediatric nurses with children. J Nurs Sci. (2025) 40:52–54+73. doi: 10.3870/j.issn.1001-4152.2025.18.052

[13] Lee K Cha H. Development and validation of an instrument for measuring parenting stress among clinical nurses. Asian Nurs Res. (2021) 15:223–30. doi: 10.1016/j.anr.2021.07.001, 34303010

[14] Zeng MQ Sun HM Huang RR Wang M Yan XX Li CL. Chinese adaptation and reliability and validity testing of the clinical nurses' parenting stress scale. Chin Nurs Manag. (2023) 23:844–8. doi: 10.3969/j.issn.1672-1756.2023.06.009

[15] Yang Q Zhao A Lee C Wang X Vorderstrasse A Wolever RQ. Latent profile/class analysis identifying differentiated intervention effects. Nurs Res. (2022) 71:394–403. doi: 10.1097/NNR.0000000000000597 35417442

[16] Liu F Yang D Liu Y Zhang Q Chen S Li W . Use of latent profile analysis and k-means clustering to identify student anxiety profiles. BMC Psychiatry. (2022) 22:12. doi: 10.1186/s12888-021-03648-7, 34986837 PMC8728926

[17] VON Elm E Altman DG Egger M Pocock SJ Gøtzsche PC Vandenbroucke JP. The strengthening the reporting of observational studies in epidemiology (STROBE) statement: guidelines for reporting observational studies. Lancet. (2007) 370:1453–7. doi: 10.1016/s0140-6736(07)61602-x 18064739

[18] Nylund-Gibson K Garber AC Carter DB Chan M Arch DAN Simon O . Ten frequently asked questions about latent transition analysis. Psychol Methods. (2023) 28:284–300. doi: 10.1037/met0000486, 35834194

[19] Saccenti E Timmerman ME. Approaches to sample size determination for multivariate data: applications to PCA and PLS-DA of omics data. J Proteome Res. (2016) 15:2379–93. doi: 10.1021/acs.jproteome.5b01029, 27322847

[20] van de Schoot R. Latent trajectory studies: the basics, how to interpret the results, and what to report. Eur J Psychotraumatol. (2015) 6:27514. doi: 10.3402/ejpt.v6.27514 PMC4348410 25735413

[21] Yao X Wen S Song Z Wang J Shen Y Huang X. Work-family conflict profiles and support strategies for married female nurses: a latent profile analysis. Front Public Health. (2024) 12:1324147. doi: 10.3389/fpubh.2024.1324147 PMC10958783 38525344

[22] Nakagawa A Hara Y Matsunaga A Nakano K Iwamoto M Sumikawa Tsuno Y . Personal growth among female nurses raising preschool children: a cross-sectional study on the role of work engagement and social support. BMC Nurs. (2025) 24:1283. doi: 10.1186/s12912-025-03941-w, 41088140 PMC12522314

[23] Crowell JA Keluskar J Gorecki A. Parenting behavior and the development of children with autism spectrum disorder. Compr Psychiatry. (2019) 90:21–9. doi: 10.1016/j.comppsych.2018.11.007, 30658339

[24] Ho LLK Li WHC Cheung AT Luo Y Xia W Chung JOK. Impact of poverty on parent-child relationships, parental stress, and parenting practices. Front Public Health. (2022) 10:849408. doi: 10.3389/fpubh.2022.849408 PMC9081330 35548071

[25] Shan Z Li Y Zong G Guo Y Li J Manson JE . Rotating night shift work and adherence to unhealthy lifestyle in predicting risk of type 2 diabetes: results from two large US cohorts of female nurses. BMJ. (2018) 363:k4641. doi: 10.1136/bmj.k4641, 30464025 PMC6247172

[26] Shi H Huang T Schernhammer ES Sun Q Wang M. Rotating night shift work and healthy aging after 24 years of follow-up in the nurses' health study. JAMA Netw Open. (2022) 5:e2210450. doi: 10.1001/jamanetworkopen.2022.10450, 35507343 PMC9069254

[27] Weaver SH De Cordova PB Vitale TR Hargwood P Salmond S. Experiences and perceptions of nurses working night shift: a qualitative systematic review. JBI Evid Synth. (2023) 21:33–97. doi: 10.11124/jbies-21-00472, 35975311

[28] Feng HL Qi XX Xia CL Feng H‐l Qi X‐x Xia C‐L . Association between night shift and sleep quality and health among Chinese nurses: a cross-sectional study. J Nurs Manag. (2021) 29:2123–31. doi: 10.1111/jonm.13351 33908108

[29] Xu X Hanafi Z Zhang S. How is parenting stress related to parental burnout among children's mothers in China: the mediating role of marital satisfaction and the moderating role of socioeconomic status. Front Public Health. (2024) 12:1431598. doi: 10.3389/fpubh.2024.1431598, 39717030 PMC11664264

[30] Yang J Gao Y Wang W Wang J Wang Y Yuan L. Interventions to address parenting stress among caregivers of children with chronic diseases: an umbrella review. J Clin Nurs. (2024) 33:1626–46. doi: 10.1111/jocn.17060, 38323676

[31] Zhou T Luo Y Xiong W Meng Z Zhang H Zhang J. Problem-solving skills training for parents of children with chronic health conditions: a systematic review and meta-analysis. JAMA Pediatr. (2024) 178:226–36. doi: 10.1001/jamapediatrics.2023.5753 PMC10762633 38165710

[32] Ong P Cong X Yeo Y Shorey S. Experiences of nurses managing parenthood and career: a systematic review and meta-synthesis. Int Nurs Rev. (2024) 71:610–25. doi: 10.1111/inr.12885, 37724722

[33] Si X Xue H Song X Liu X Zhang F. The relationship between ethical leadership and nurse well-being: the mediating role of workplace mindfulness. J Adv Nurs. (2023) 79:4008–21. doi: 10.1111/jan.15719 37226654

[34] Zhu Y Zhang Y Qin F Li Y. The relationships of leaders' narcissistic admiration and rivalry with nurses' organizational citizenship behavior towards leaders: a cross-sectional survey. J Nurs Manag. (2023) 2023:1–8. doi: 10.1155/2023/5263017, 40225621 PMC11919070

